# Metabolomics and transcriptomics to decipher molecular mechanisms underlying ectomycorrhizal root colonization of an oak tree

**DOI:** 10.1038/s41598-021-87886-5

**Published:** 2021-04-21

**Authors:** M. Sebastiana, A. Gargallo-Garriga, J. Sardans, M. Pérez-Trujillo, F. Monteiro, A. Figueiredo, M. Maia, R. Nascimento, M. Sousa Silva, A. N. Ferreira, C. Cordeiro, A. P. Marques, L. Sousa, R. Malhó, J. Peñuelas

**Affiliations:** 1 Plant Functional Genomics Group, BioISI – Instituto de Biosistemas e Ciências Integrativas, Faculdade de Ciências, Universidade de Lisboa, Lisbon, Portugal; 2 Global Change Research Institute of the Czech Academy of Sciences, Bělidla 986/4a, CZ-60300 Brno, Czech Republic; 3 CREAF, 08193 Cerdanyola del Vallès, Catalonia Spain; 4 CSIC, Global Ecology Unit CREAF-CSIC-UAB, 08193 Bellaterra, Catalonia Spain; 5 Servei de Ressonàcia Magnètica Nuclear, Universitat Autònoma de Barcelona, 08193 Cerdanyola del Vallès, Catalonia Spain; 6 Centre for Ecology, Evolution and Environmental Changes (CE3C), Faculdade de Ciências, Universidade de Lisboa, Lisbon, Portugal; 7 Linking Landscape, Environment, Agriculture and Food (LEAF), Instituto Superior de Agronomia (ISA), Universidade de Lisboa, Lisbon, Portugal; 8 Laboratório de FTICR e Espectrometria de Massa Estrutural, Departamento de Química e Bioquímica, Faculdade de Ciências, Universidade de Lisboa, Lisbon, Portugal; 9 CEAUL - Centro de Estatística e Aplicações, Faculdade de Ciências, Universidade de Lisboa, Lisbon, Portugal; 10 Spanish National Research Council, Madrid, Spain

**Keywords:** Molecular biology, Plant sciences

## Abstract

Mycorrhizas are known to have a positive impact on plant growth and ability to resist major biotic and abiotic stresses. However, the metabolic alterations underlying mycorrhizal symbiosis are still understudied. By using metabolomics and transcriptomics approaches, cork oak roots colonized by the ectomycorrhizal fungus *Pisolithus tinctorius* were compared with non-colonized roots. Results show that compounds putatively corresponding to carbohydrates, organic acids, tannins, long-chain fatty acids and monoacylglycerols, were depleted in ectomycorrhizal cork oak colonized roots. Conversely, non-proteogenic amino acids, such as gamma-aminobutyric acid (GABA), and several putative defense-related compounds, including oxylipin-family compounds, terpenoids and B6 vitamers were induced in mycorrhizal roots. Transcriptomic analysis suggests the involvement of GABA in ectomycorrhizal symbiosis through increased synthesis and inhibition of degradation in mycorrhizal roots. Results from this global metabolomics analysis suggest decreases in root metabolites which are common components of exudates, and in compounds related to root external protective layers which could facilitate plant-fungal contact and enhance symbiosis. Root metabolic pathways involved in defense against stress were induced in ectomycorrhizal roots that could be involved in a plant mechanism to avoid uncontrolled growth of the fungal symbiont in the root apoplast. Several of the identified symbiosis-specific metabolites, such as GABA, may help to understand how ectomycorrhizal fungi such as *P. tinctorius* benefit their host plants.

## Introduction

Most land plants live in association with specialized soil-born fungi that colonize their roots forming mycorrhizae, the most widespread plant-symbiotic relationship. At the centre of this symbiosis is the mutual exchange of nutrients between partners: the fungus provides mineral nutrients to the plant, that in turn transfers to the fungus sugars produced during photosynthesis^1^. Temperate forest trees, such as Pinus, Oaks and Eucalyptus form a distinct type of mycorrhizas, called ectomycorrhizas (ECM), with Basidiomycotic and Ascomycotic fungi, such as boletes and truffles. In ECMs, the fungal mycelium colonizes the apoplast of root cells forming a net of hyphae that surrounds the epidermal and/or the cortex cells. By also spreading into the soil, the fungal mycelium can access mineral nutrients very efficiently, such as nitrogen and phosphorous, which are then transferred to the root, ECM greatly increasing the absorptive surface area of the host plant root system. However, ECM symbiosis can also help plants to increase resistance to multiple stresses. Indeed, several studies point to a better performance of ECM plants against abiotic and biotic stress, such as drought, soil salinity, heavy metal contamination and protection from pathogens^2–5^. Therefore, there is an increasing interest in using mycorrhizas to enhance the resilience of plants to the increasingly stressful environmental conditions and to aid in the desired reduction of fungicide and fertilizer application. Thus, studies on the molecular basis of mycorrhization are fundamental to be able to use the advantages of this plant symbiotic interactions, either by simple inoculation or by more complex approaches such as genome editing to copy mycorrhizal benefits to crop plants.

Cork oak (*Quercus suber*) is an evergreen tree species found in the western part of the Mediterranean basin where it is exploited for the production of cork, the thick outer bark layer that covers the trunk and can be extracted periodically without any harm to the tree, which regenerates back a new cork layer that can be peeled again from the tree. This sustainable agro-forestry system, with low human intervention is characterized by a high biodiversity of both plant and animal species (https://forest.jrc.ec.europa.eu/en/european-atlas/). However, cork oak forests are threatened by fire, agricultural expansion, diseases and climate change. Therefore, the plantation of new areas with cork oak is mandatory and could benefit from the use of mycorrhizal seedlings to increase survival of new plantations in the hot and dry regions of the western Mediterranean region.

This study aims to provide a comprehensive view of the metabolic alterations occurring in roots upon ECM symbiosis establishment using the cork oak – *Pisolithus tinctorius* ECM interaction as a model. Both partners efficiently form ECMs in greenhouse conditions, and have been the subject of transcriptomics and proteomics studies by our team, that have revealed some important molecular aspects of the regulation and metabolism of their symbiotic relationship^6,7^.

Currently, metabolomics technologies allow the separation and identification of a wide range of metabolites providing a global overview of the qualitative and quantitative changes of plant primary and secondary metabolites^8–11^. However, few studies have addressed metabolic alterations underlying ECM symbiosis^2,12–14^. Previous untargeted metabolomics studies have shown a strong effect of ECM symbiosis on primary and secondary poplar root metabolism, mainly on aromatic acid, organic acid and fatty acids which were induced, while sugars were reduced^12^. On the other hand, investigation of the metabolic alterations in roots of an oak tree species colonized by the ECM fungus *Tuber indicum* indicated the accumulation of carbohydrates and organic acids^13^. Studies on the pre-symbiotic phase of ECM interaction in *Eucalyptus grandis* roots have shown a symbiotic specific plant metabolic response associated to the suppression of plant metabolism, which can be crucial in determining the outcome of the interaction^14^.

Using two untargeted metabolomics technologies, Proton Nuclear Magnetic Resonance (^1^H NMR) spectroscopy and Direct Infusion Fourier-Transform Ion Cyclotron Resonance Mass Spectrometry (DI-FT-ICR-MS), we analysed the metabolome of ECM roots. We detected major metabolic alterations associated with ECM formation in the roots of cork oak, and some of them were investigated in deeper detail by gene expression analysis. The impact of the identified changes is discussed in relation to the plant-fungus ECM symbiotic program.

## Results

### Effect of *P. tinctorius* on cork oak root colonization and growth

**Figure 1:**
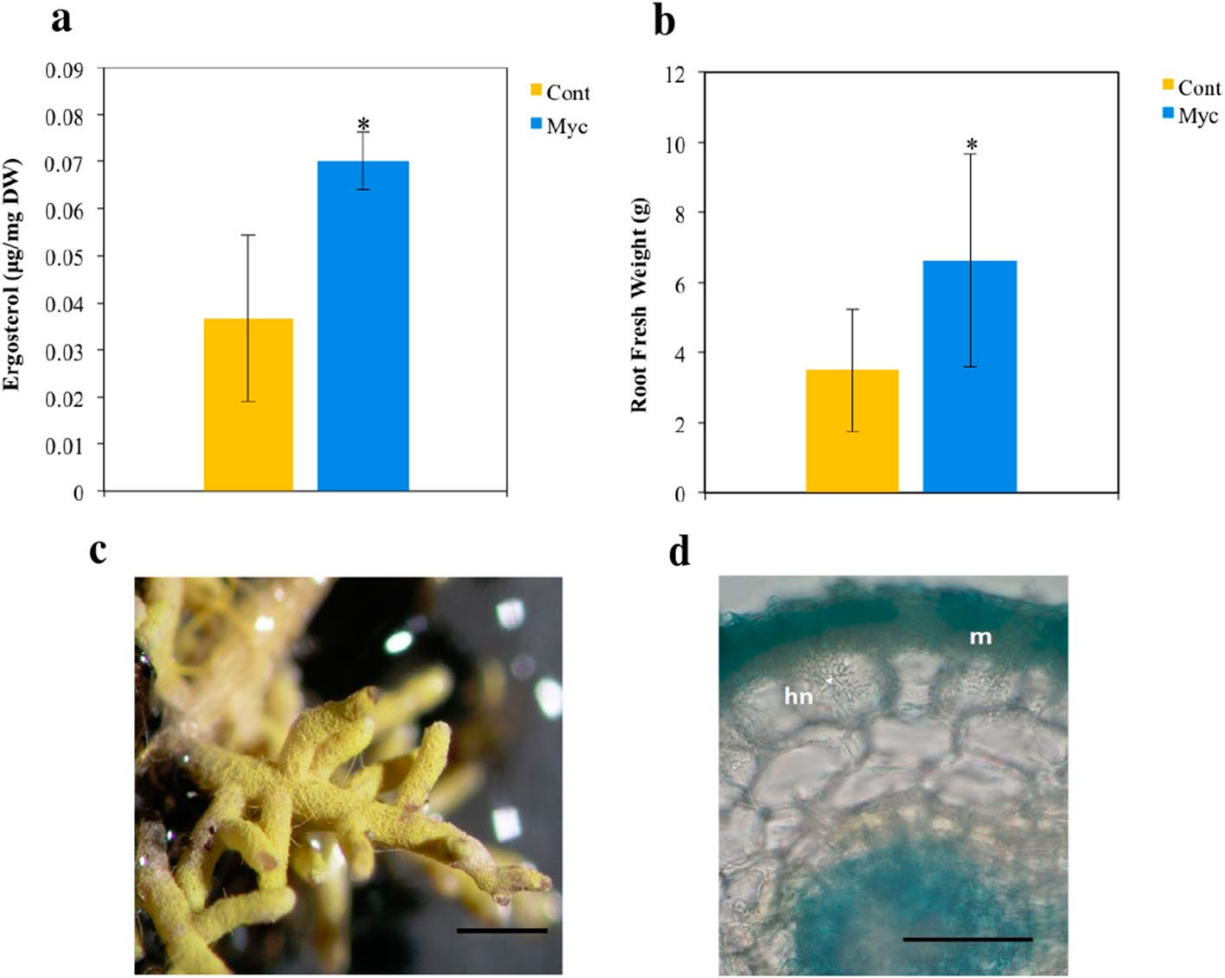
Fungal root colonization and plant growth of mycorrhizal (Myc) and non-inoculated (Cont) cork oak plants, 8 weeks after *P. tinctorius* inoculation. (**a**) Ergosterol root content. Data represent the mean of 3 independent biological replicates (each composed of a pool of 3 individual root systems) ± SD (**b**) Root fresh weight. Data represent the mean of 15 independent plants ± SD. *Indicates statistical differences at the level *P* < 0.05 [t-test for (**a**) and Man-Whitney *U* test for (**b**)]. (**c**) Colonized root showing the hyphal mantle. Scale bar = 1 mm. (**d**) Representative trypan blue staining of a colonized root showing the fungal mantle (m) and the hartig net (hn). Scale bar = 50 μm.

The ergosterol concentration was significantly higher in *P. tinctorius* inoculated roots relative to the non-inoculated plants, indicating the formation of a functional ECM symbiosis in cork oak roots upon inoculation (Fig. 1a). It is known that ECM symbiosis can have a positive effect on the growth and nutrient acquisition of colonized plants^1^. *P. tinctorius* inoculation had a positive effect on root growth, significantly increasing root biomass (*P* < 0.05), with mycorrhizal plants showing 1.89 times more root fresh weight than non-inoculated plants (Fig. 1b). Leaf biomass and root N concentration were not significantly affected by the *P. tinctorius* inoculation (Supplementary Table S1). Microscopic observations of the inoculated roots confirmed the ECM colonization of the cork oak roots by *P. tinctorius*. Typical ECM structures, such as the fungal mantle and the Hartig net were clearly observed in roots, 8 weeks after *P. tinctorius* inoculation (Fig. 1c,d). The level of *P. tinctorius* biomass (estimated by the concentration of ergosterol) in mycorrhizal roots was found to be 7% (Supplementary Table S1).

### *P. tinctorius* colonization induces major metabolic changes in cork oak roots

**Figure 2:**
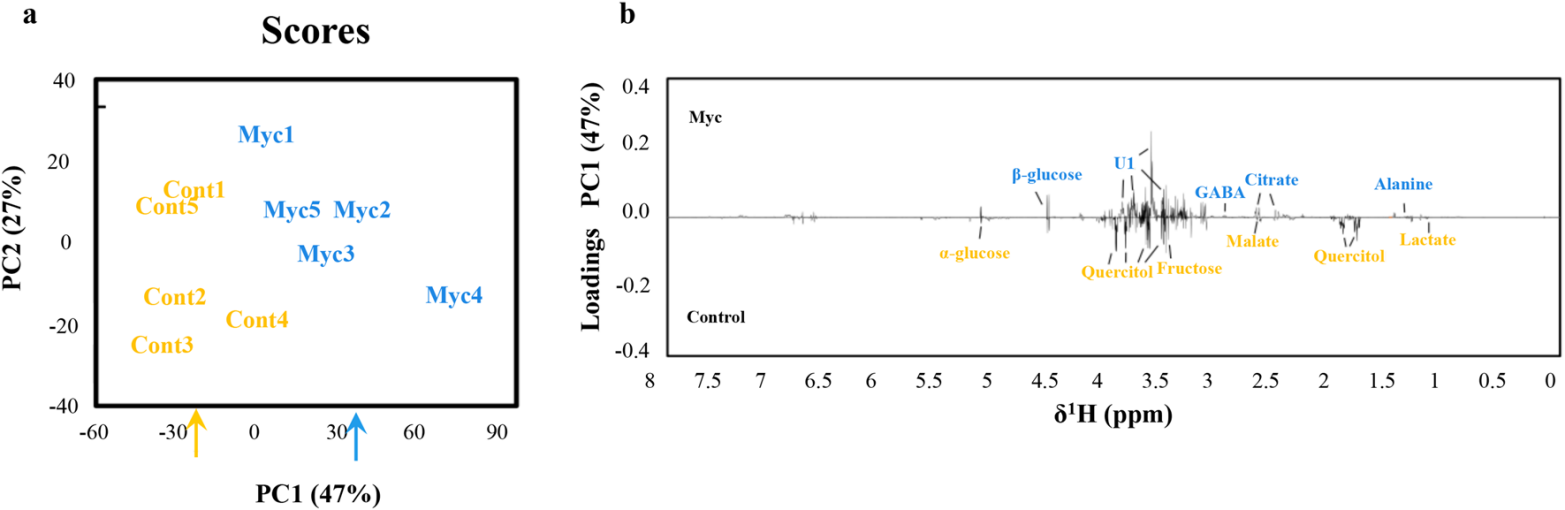
Overview of the metabolic reprograming (analysed by 1H NMR) in mycorrhizal (Myc) and non-inoculated (Cont.) roots interpreted using PCA. (**a**) Biplot of the second principal component (PC2) versus the first principal component (PC1) scores. (**b**) Loading values of the first principal component (PC1). The molecular name of the elucidated peaks with significantly different concentration in mycorrhizal compared to non-inoculated roots is highlighted; blue corresponds to metabolites with higher concentrations in mycorrhizal roots and orange corresponds to those with higher concentration in non-inoculated roots.

**Figure 3:**
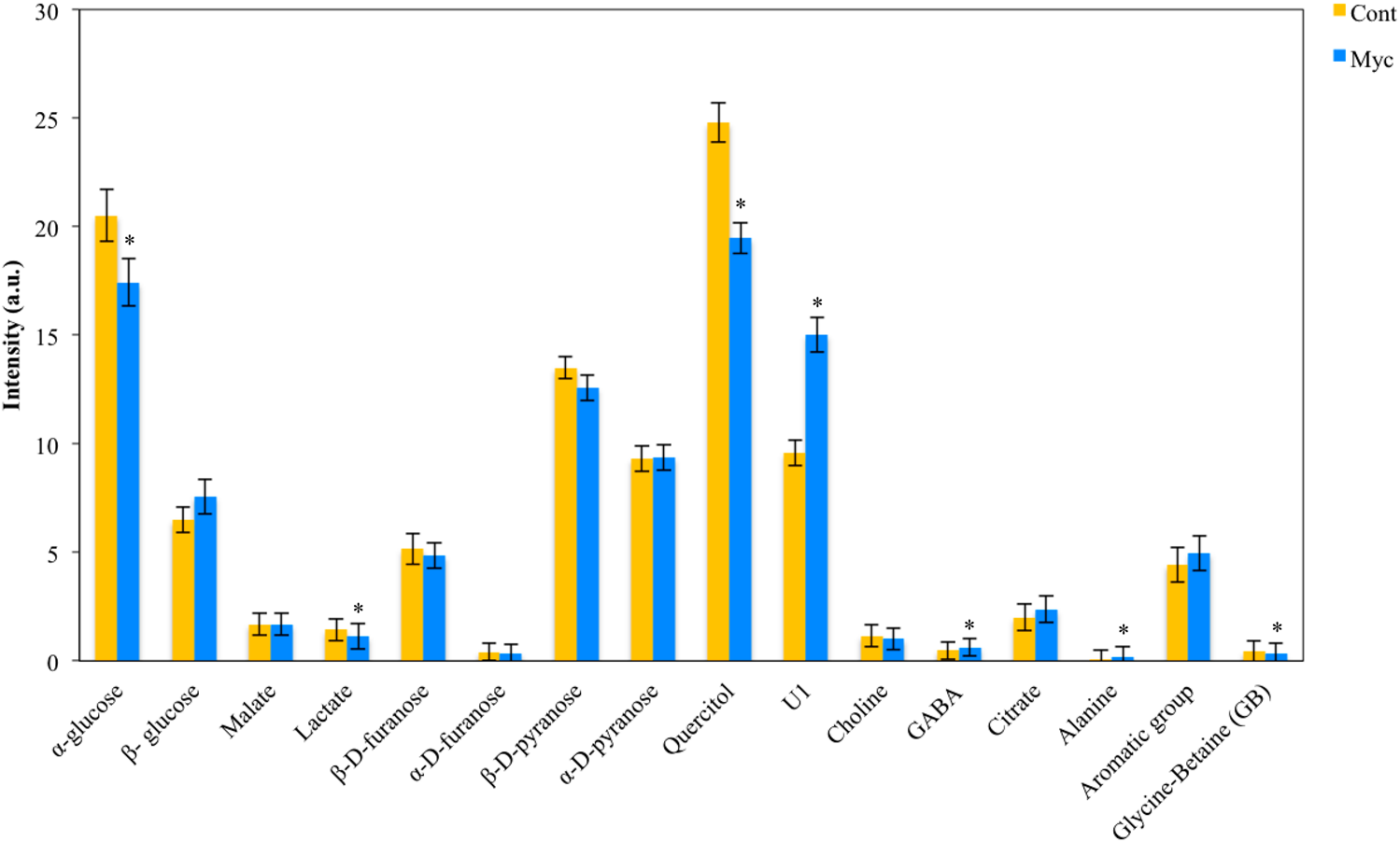
Comparisons between mycorrhizal (Myc) and non-inoculated (Cont) peak areas of root metabolites identified by NMR analysis. *Indicates statistical differences at the level *P* < 0.05 (t-test).

NMR analysis of mycorrhizal and non-inoculated roots enabled the identification of 15 different metabolites (Supplementary Table S2 and Supplementary Fig. S1). A principal component analysis (PCA) of the different metabolites as variables and the different treatments as cases, showed a clear separation between mycorrhizal and non-inoculated roots suggesting a different metabolic response (Fig. 2a). Inoculation with *P. tinctorius* had a strong effect on several individual peaks that presented significant differences between mycorrhizal and non-inoculated roots (*P* < 0.05) (Figs. 2b, 3). Mycorrhizal roots showed higher levels of the amino acid’s GABA and alanine, the sugar β-glucose, the organic acid citrate and the aromatic unknown compound 1. In contrast, the organic osmolytes quercitol (a polyol) and glycine-betaine, the sugars α-glucose and fructose, and the organic acids malate and lactate, showed decreased levels in mycorrhizal roots.

**Figure 4:**
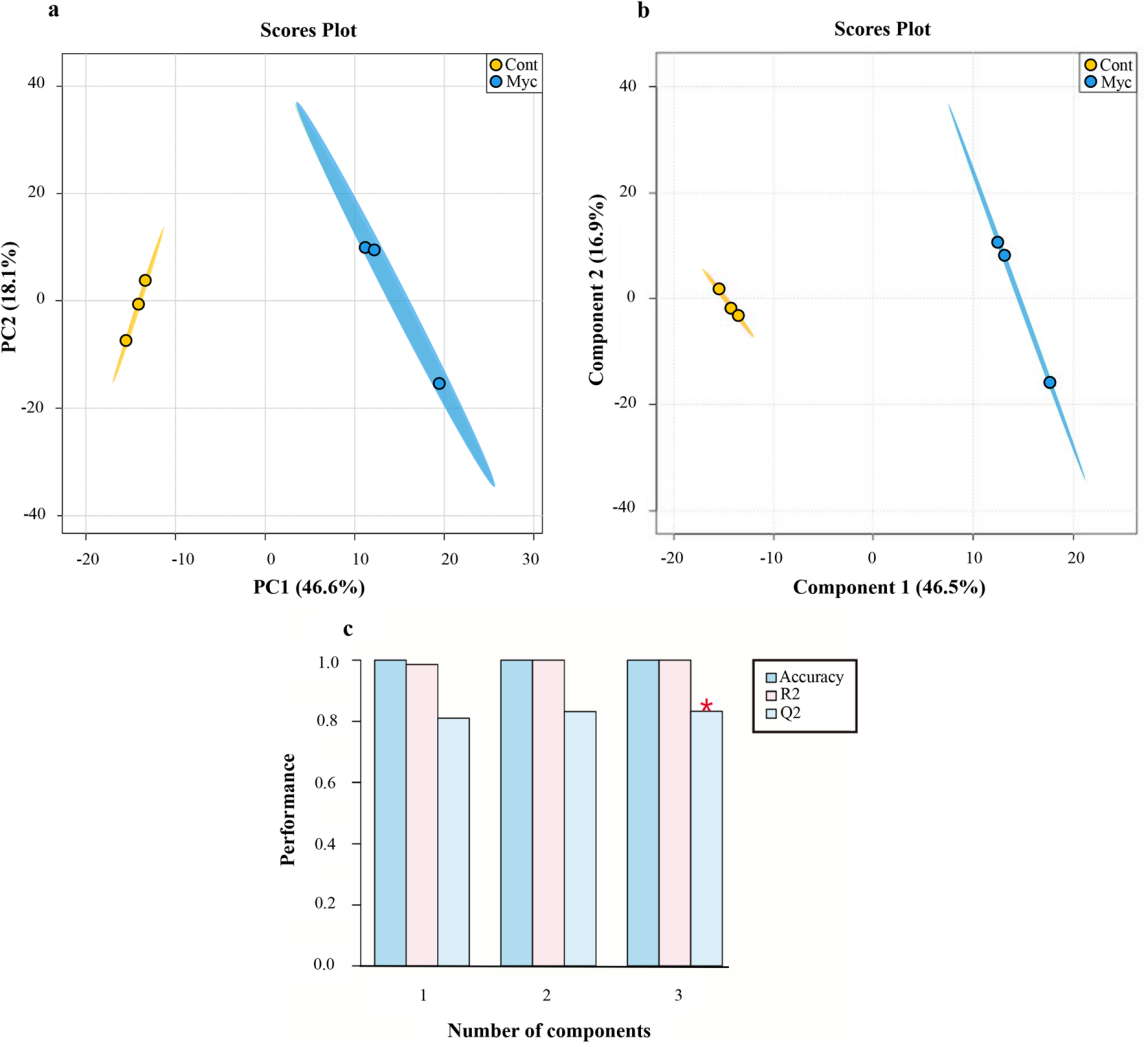
Overview of the metabolic reprograming analysed by FT-ICR in mycorrhizal and non-inoculated roots interpreted using PCA and PLS-DA. (**a**) PCA scores plot of mycorrhizal (Myc) and non-inoculated (Cont) cork oak roots. Each point represents a biological replicate (**b**) PLS-DA scores plot of mycorrhizal (Myc) and non-inoculated (Cont) cork oak roots. Each point represents a biological replicate. (**c**) Cross-validation scores plot of the PLS-DA for the classification into mycorrhizal and non-inoculated groups using the FT-ICR data as a function of the number of PLS components. The number of components that maximizes Q2 is indicated with a star. Charts were produced with the free available on-line software MetaboAnalyst v4.0 (https://www.metaboanalyst.ca/).

**Figure 5:**
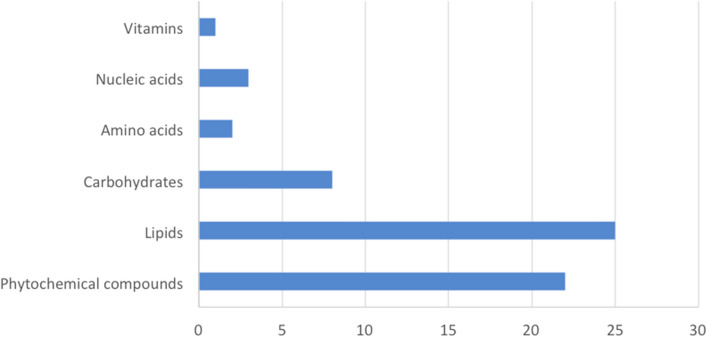
Chemical classes associated with the annotated metabolites discriminating mycorrhizal roots from non-inoculated roots (VIP > 1) analysed by FT-ICR. The x axis corresponds to the number of annotated masses assigned to each class**.**

**Figure 6:**
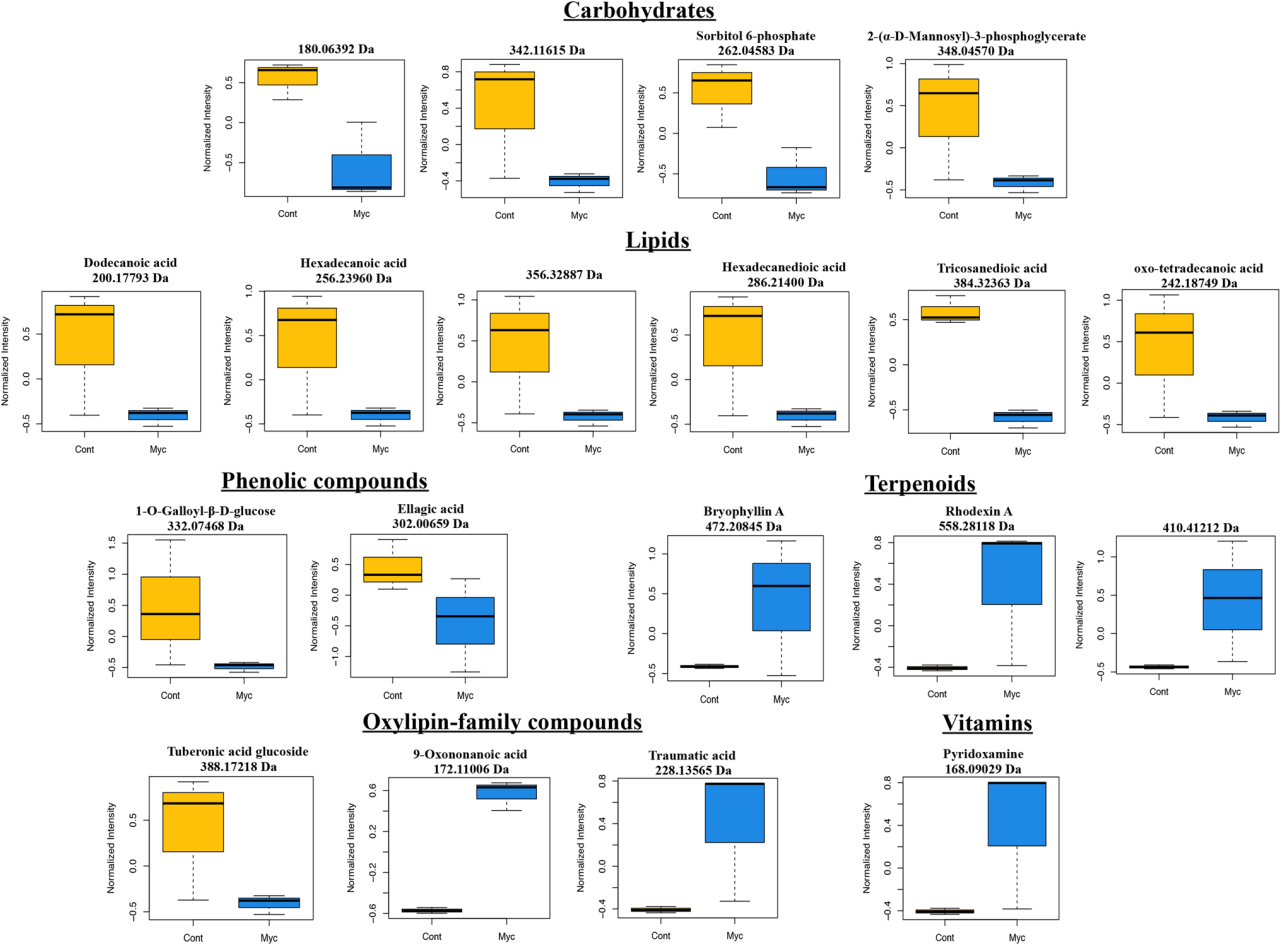
Accumulation profile of selected metabolites (VIP > 1) in cork oak mycorrhizal roots. *P. tinctorius* inoculated roots (Myc) and non-inoculated roots (Cont) were used for relative quantification by FT-ICR analysis. Compounds only tentatively identified are identified by an *m/z* ratio. Charts were produced with the free available on-line software RStudio v1.2.5042^83^.

The FT-ICR analysis of the two metabolite fractions of mycorrhizal and non-inoculated roots revealed a mean number of about 4400 peaks per replicate in ESI + mode and 400 in ESI – ionization modes (Supplementary Table S3). The van Krevelen diagrams representing elemental formulas of mycorrhizal and non-inoculated roots revealed the high chemical diversity present in those samples (Supplementary Fig. S2). Notable differences between treatments were detected, with mycorrhizal roots showing fewer elemental formulas, specifically in areas corresponding to polyketides and carbohydrates. PCA analysis showed clear metabolic differences between mycorrhizal and non-inoculated roots (Fig. 4a), with the first two PCs explaining 64.7% of the total variance of the data, suggesting that both PC’s are sufficient to discriminate between the inoculation treatments (mycorrhizal and non-inoculated). To access which of the metabolites are most responsible for the observed differences that correlated with *P. tinctorius* inoculation, we performed a supervised analysis using PLS-DA and used VIP scores to establish the importance of mass features. The scores plot showed a clear separation between mycorrhizal and non-inoculated groups (Fig. 4b), suggesting a separated root metabolite profile corresponding to each treatment. Internal cross-validation showed a value of Q^2^ above 0.8, indicative of a good predictive performance of the PLS-DA model, with a maximum Q^2^ achieved with 3 PLS components (Fig. 4c). Masses with the highest discriminative capacity (VIP score > 1) were selected as the most important metabolites that contributed for the separation of the metabolic changes identified. The VIP score describes a quantitative estimation of the discriminatory power of each individual feature^15^ and VIP > 1 is commonly used as a cut-off for selecting the most important features separating the defined classes of samples in PLS-DA^14,16,17^. Using the MassTrix interface we were able to putatively annotate 61 metabolites discriminating between the inoculation treatments, including 37 with decreased levels, and 24 with increased levels in mycorrhizal roots compared to non-inoculated controls (Table 1; Supplementary Table S4). Classification of these metabolites into chemical classes revealed that inoculation with *P. tinctorius* affected 6 major classes of compounds: phytochemical compounds, lipids, carbohydrates, amino acids, nucleic acids and vitamins (Fig. 5). Mycorrhizal colonization had a strong effect on root primary metabolism, mainly on metabolites putatively corresponding to lipids and carbohydrates, including many putative fatty acids and monosaccharides, but also affected secondary metabolism, altering some phytochemical compounds, such as putative alkaloids, terpenoids and oxylipin-related compounds (Table 1; Supplementary Table S4). Among the differentially accumulated metabolites involved in primary metabolism, just like in the NMR analysis, several putative carbohydrates showed decreased levels in mycorrhizal roots, including isomers of glucose (180.06392 Da) and sucrose (342.11615 Da), and also of other carbohydrates related to the biosynthesis of sugar alcohols (polyols), such as sorbitol (sorbitol 6-phosphate, 262.04583 Da) and manosylglycerate (2-(α-D-mannosyl)-3-phosphoglycerate, 348.04570 Da) (Fig. 6; Table 1; Supplementary Table S4).

**Table 1:** Putative identities of the metabolites discriminating mycorrhizal (myc) roots from non-inoculated (cont) roots analyzed by FT-ICR.

Neutral mass (Da)	Putative compound identity	Major class	Secondary class	VIP Score*	Log2 (FC) myc vs cont
436.25858	LPA (0:0/18:1(9Z))	Lipids	Glycerophospholipids	1.044	− 2.426
424.29647	LPA (18:0e/0:0)	Lipids	Glycerophospholipids	1.024	2.100
246.05016	Glycerophosphoglycerol	Lipids	Glycerophospholipids	1.143	2.998
369.32455	N-stearoyl GABA#N-palmitoyl isoleucine#N-palmitoyl leucine	Lipids	Fatty amides	1.007	− 2.169
341.29252	Stearoylglycine#N-palmitoyl GABA	Lipids	Fatty amides	1.086	2.402
283.28694	Octadecanamide	Lipids	Fatty amides	1.038	− 2.225
383.34046	N-stearoyl valine	Lipids	Fatty amides	1.004	2.534
281.27209	Oleamide	Lipids	Fatty amides	1.070	− 2.589
248.17698	16:4(4Z,7Z,10Z,13Z)	Lipids	Fatty acids	1.469	2.729
340.33514	Docosanoic acid	Lipids	Fatty acids	1.121	2.742
270.25520	Palmitic acid methyl ester#Margaric acid#( +)-14-methyl palmitic acid#15-methyl palmitic acid	Lipids	Fatty acids	1.092	2.454
284.27080	Octadecanoic acid (Stearic acid)#( +)-Isostearic acid	Lipids	Fatty acids	1.047	2.229
200.17793	Dodecanoic acid	Lipids	Fatty acids	1.016	− 2.174
298.28656	Nonadecanoic acid#16-methyl-octadecanoic acid#17-methyl-octadecanoic acid#11-methyl-octadecanoic acid	Lipids	Fatty acids	1.023	− 2.121
256.23960	Hexadecanoic acid (palmitic acid)#Isopalmitic acid#13-methyl-pentadecanoic acid	Lipids	Fatty acids	1.006	− 2.138
356.32887	13-Hydroxydocosanoic acid#2-Hydroxydocosanoic acid#22-Hydroxydocosanoic acid	Lipids	Fatty acids	1.058	− 2.386
384.32363	Tricosanedioic acid	Lipids	Fatty acids	1.449	− 2.549
286.21400	Hexadecanedioic acid	Lipids	Fatty acids	1.019	− 2.193
242.18749	3-oxo-tetradecanoic acid#6-oxo-tetradecanoic acid	Lipids	Fatty acids	1.040	− 2.396
128.05880	gamma-Amino-gamma-cyanobutanoate#alpha-Amino-gamma-cyanobutanoate	Lipids	Fatty acids	1.007	− 2.133
358.30764	MG(18:0/0:0/0:0)#MG(0:0/18:0/0:0)	Lipids	Monoacylglycerols	1.764	− 4.584
358.30797	MG(0:0/18:0/0:0)# MG(18:0/0:0/0:0)	Lipids	Monoacylglycerols	1.403	− 2.454
330.27638	MG(0:0/16:0/0:0)#MG(16:0/0:0/0:0)	Lipids	Monoacylglycerols	1.012	− 2.050
302.24490	MG(0:0/14:0/0:0)#MG(14:0/0:0/0:0)	Lipids	Monoacylglycerols	1.129	− 1.435
250.11981	Methylripariochromene A#Ubiquinone Q1	Lipids	Prenol lipids	1.032	− 2.330
347.07876	Hydroxysanguinarine	Phytochemical compounds	Alkaloids	1.549	3.145
354.23015	Aspidospermine	Phytochemical compounds	Alkaloids	1.519	− 2.863
315.14751	3′-Hydroxy-N-methyl-(S)-coclaurine#(R)-Norreticuline#(S)-Norreticuline#Cephalotaxine	Phytochemical compounds	Alkaloids	1.459	− 2.540
305.16269	(6S)-Hydroxyhyoscyamine#Porritoxin#Lunacridine#Anisodamine#Convolamine	Phytochemical compounds	Alkaloids	1.129	2.960
240.14751	Slaframine	Phytochemical compounds	Alkaloids	1.054	2.165
398.22030	Desacetoxyvindoline#Mitragynine	Phytochemical compounds	Alkaloids	1.008	− 2.177
385.15301	Polycarpine	Phytochemical compounds		1.010	2.028
430.21168	Cinegalline	Phytochemical compounds	Alkaloids	1.089	− 2.675
287.24570	Prosopinine	Lipids	Alkaloids	1.040	− 2.240
267.13732	Ergine	Phytochemical compounds	Alkaloids	1.007	− 2.028
332.07468	1-O-Galloyl-beta-D-glucose	Phytochemical compounds	Phenolic compounds	1.201	− 4.247
302.00659	Ellagic acid	Phytochemical compounds	Phenolic compounds	1.099	− 1.782
310.15629	Dihydrocordoin	Phytochemical compounds	Flavonoids	1.066	2.362
298.08401	Apigenin 7,4′-dimethyl ether#Afrormosin#Sayanedine#Sayanedine#Cladrin#Afrormosin#Alfalone#8-O-Methylretusin#5-O-Methylbiochanin A#Pterocarpin#Kuhlmannin#4′-Hydroxy-5,7-dimethoxy-4-phenylcoumarin#Tithonine#7-Hydroxy-3′,4′-dimethoxyflavone#5-Hydroxy-6,2′-dimethoxyflavone#5-Hydroxy-7,2′-dimethoxyflavone#Syzalterin#8-Demethylsideroxylin#Apigenin 7,4′-dimethyl ether#Baicalein 5,6-dimethyl ether#Mosloflavone#7-Hydroxy-5,8-Dimethoxyflavone#7-O-Methylwogonin#5,7-Dihydroxy-3-methoxy-8-methylflavone#3,5-Dihydroxy-7-methoxy-8-methylflavone#3,5,7-Trihydroxy-6,8-dimethylflavone#3,7-Dimethoxy-5-hydroxyflavone#Galangin 5,7-dimethyl ether#Isoneobavachalcone#Neobavachalcone#Lawinal	Phytochemical compounds	Flavonoids	1.012	2.034
472.20845	Bryophyllin A	Phytochemical compounds	Terpenoids	1.019	2.663
558.28118	Rhodexin A	Phytochemical compounds	Terpenoids	1.009	1.925
410.41212	(S)-2,3-Epoxysqualene#LanosterolCycloartenol#Obtusifoliol#Cycloeucalenol#alpha-Amyrin#beta-Amyrin#Euphol#Friedelin#Lupeol#Taraxasterol#Taraxerol#24-Ethylidene lophenol#Fernenol#Isoarborinol#( +)-Tirucallol	Lipids#Phytochemical compounds	Terpenoids	1.076	2.663
228.13565	Traumatic acid	Phytochemical compounds	Oxylipins	1.008	2.019
172.11006	9-Oxononanoic acid	Phytochemical compounds	Oxylipins	1.414	2.422
388.17218	Tuberonic acid glucoside	Phytochemical compounds	Oxylipins	1.015	− 2.071
208.04069	2-(2′-Methylthio)ethylmalic acid#3-(2′-Methylthio)ethylmalic acid	Phytochemical compounds	Glucosinolates	1.489	2.826
339.10701	Vulgaxanthin-I	Phytochemical compounds	Amino acid related compounds	1.012	− 2.052
180.06392	D-Glucose#alpha-D-Glucose#beta-D-Glucose#beta-D-Glucoside#D-Fructose#L-Fructose#beta-D-Fructose#Galactose#D-Galactose#L-Galactose#alpha-D-Galactose#myo-Inositol#scyllo-Inositol#D-Mannose#L-Sorbose#D-Sorbose#D-Tagatose#D-Allose#L-Fuconate#L-Rhamnonate#D-Altrose#D-Gulose#D-Idose#D-Talose#D-Psicose#L-Gulose#Scyllitol	Carbohydrates	Monosaccharides	1.375	− 2.362
179.07992	D-Glucosamine#beta-D-Glucosamine	Carbohydrates	Monosaccharides	1.028	2.169
262.04583	Sorbitol 6-phosphate	Carbohydrates		1.298	− 2.344
307.09057	S-Succinyldihydrolipoamide-E	Carbohydrates		1.015	2.568
290.03955	Sedoheptulose 7-phosphate	Carbohydrates		1.039	1.346
348.04570	2-(alpha-D-Mannosyl)-3-phosphoglycerate	Carbohydrates		1.034	− 2.198
342.11615	Sucrose#Cellobiose#Maltose#Lactose#Isomaltose#Trehalose#Galactinol#Nigerose#Mannobiose#Palatinose#Laminaribiose#Melibiose#Turanose	Carbohydrates	Oligosaccharides	1.012	− 2.053
260.03017	D-Fructose 6-phosphate#D-Fructose 1-phosphate#beta-D-Fructose 2-phosphate#beta-D-Fructose 6-phosphate#D-Glucose 6-phosphate#D-Glucose 1-phosphate#beta-D-Glucose 1-phosphate#alpha-D-Glucose 6-phosphate#beta-D-Glucose 6-phosphate#D-Mannose 6-phosphate#D-Mannose 1-phosphate#D-Galactose 6-phosphate#alpha-D-Galactose 1-phosphate#D-Galactose 1-phosphate#L-Galactose 1-phosphate#L-Galactose 1-phosphate#Inositol 1-phosphate#myo-Inositol 4-phosphate#1D-myo-Inositol 3-phosphate#D-Myo-inositol 4-phosphate#D-arabino-3-Hexulose 6-phosphate#L-Gulose 1-phosphate#Dolichyl phosphate D-mannose#D-Tagatose 6-phosphate#Sorbose 1-phosphate#D-Allose 6-phosphate	Carbohydrates		1.010	− 1.679
196.03657	3-(3,4-Dihydroxyphenyl) pyruvate	Amino acids		1.061	− 2.413
110.04826	Imidazole-4-acetaldehyde	Amino acids		1.026	− 2.254
288.05985	Dihydrouracil	Nucleic acids		1.049	− 2.433
308.04092	Uridine monophosphate#Pseudouridine 5′-phosphate#Uridine 3′-monophosphate#Orotidine	Nucleic acids	Nucleotides	1.040	− 2.335
114.04325	Deoxyuridine monophosphate	Nucleic acids	Nucleotides	1.018	− 2.088
168.09029	Pyridoxamine	Vitamins and Cofactors	Vitamins	1.005	2.004

*VIP > 1 indicates important features discriminating Myc from Cont treatments.

Unlike what was found using NMR, which revealed increased levels of certain amino acids in mycorrhizal roots, metabolites putatively involved in amino acid metabolism detected by FT-ICR were found to be mostly less abundant in mycorrhizal roots, including compounds putatively involved in tyrosine metabolism (3-(3,4-dihydroxyphenyl)pyruvate) and histidine metabolism (imidazole-4-acetaldehyde) (Table 1; Supplementary Table S4).

Also, a decrease in the levels of putative fatty acids was detected in the mycorrhizal roots, including decreases in isomers of dodecanoic acid (200.17793 Da), hexadecanoic acid (256.23960 Da), hydroxydocosanoic acid (356.32887 Da), hexadecanedioic acid (286.21400 Da), tricosanedioic acid (384.32363 Da) and oxo-tetradecanoic acid (242.18749 Da) (Fig. 6; Table 1; Supplementary Table S4). Other putative lipophilic compounds also less accumulated in the mycorrhizal roots, include putative monoacylglycerols, such as MG(18:0/0:0/0:0), MG(0:0/16:0/0:0) and MG(0:0/14:0/0:0) (Table 1; Supplementary Table S4). Regarding metabolites in the secondary metabolism category, a significant decrease in putative phenolic compounds which are known to be involved in the biosynthesis of hydrolysable tannins, such as 1-O-galloyl-beta-D-glucose (332.07468 Da) and ellagic acid (302.00659 Da) was detected in mycorrhizal roots (Fig. 6; Table 1; Supplementary Table S4). Other secondary compounds matched metabolites related to plant stress response mechanisms, such as those putatively involved in the oxylipin pathway. In this context we identified decreased levels of a compound putatively corresponding to tuberonic acid glucoside (388.17218 Da) in mycorrhizal roots, while the levels of two compounds putatively corresponding to 9-oxononanoic acid (172.11006 Da) and traumatic acid (228.13565 Da) were increased (Fig. 6; Table 1; Supplementary Table S4). In addition, metabolites in the terpenoids category, showed increased levels in colonized roots, including putative terpene glycosides [e.g. bryophyllin A (472.20845 Da); rhodexin A (558.28118 Da)] and compounds putatively involved in the biosynthesis of terpenoids and steroids [e.g. (S)-2,3-epoxysqualene (410.41212 Da)] (Fig. 6; Table 1; Supplementary Table S4). Other compounds related to defense, such as putative alkaloids, were identified in high numbers in cork oak roots, some being up- but most being down-accumulated in the *P. tinctorius* mycorrhizal roots (Table 1; Supplementary Table S4). Another identified compound implicated in the interaction of plants with microorganisms, which was increased in the mycorrhizal cork oak roots was a metabolite putatively corresponding to pyridoxamine (vitamin B6) (168.09029 Da) (Fig. 6; Table 1; Supplementary Table S4).

### Evaluation of the expression of genes involved on GABA biosynthesis in mycorrhizal roots

**Figure 7:**
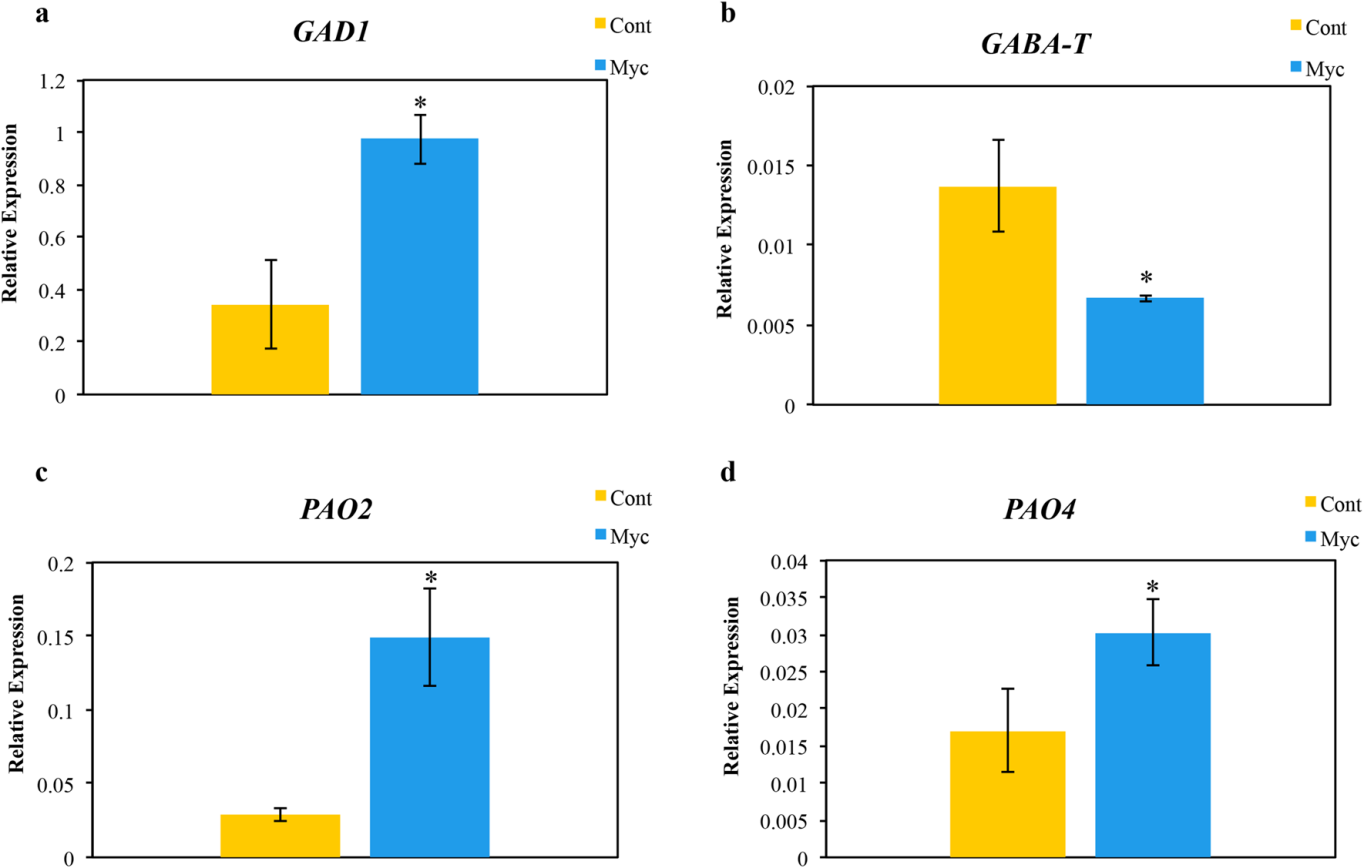
Genes expression profile of genes involved in GABA metabolism (*GAD1*, *GABAT*, *PAO2*, *PAO4*) analysed by real-time PCR. Bars represent averages ± SD of 3 biological replicates of mycorrhizal (Myc) and non-inoculated (Cont) cork oak plants. *Indicate statistical differences at the level *P* < 0.05 (Mann–Whitney *U* test).

Since our metabolomic analysis pointed to the involvement of GABA in ECM symbiosis and since recognition of pathogens and symbionts has proven to trigger GABA accumulation in several plant species^18^, we decided to investigate in more detail the molecular basis of the increased levels of GABA found in mycorrhizal roots*.* Results from the qPCR analysis (Fig. 7) show that the cork oak GAD1 transcript encoding the root specific GAD^19^ was increased in mycorrhizal roots relative to non-inoculated roots, while the expression of GABAT, which degrades GABA into succinic acid semialdehyde, was decreased. The accumulation of GABA seems to be of plant origin since transcription of the *P. tinctorius* GAD gene was barely detected in our qPCR analysis. The 2 genes encoding polyamine oxidases (PAO2 and PAO4) were increased in mycorrhizal roots, showing that GABA accumulation in cork oak mycorrhizal roots could result from polyamine catabolism (Fig. 7).

## Discussion

In our experiment, the level of ergosterol concentration of mycorrhizal roots is consistent with a highly compatible interaction between cork oak and the *P. tinctorius* fungal strain used in our analysis. Increases in root biomass, as detected in cork oak mycorrhizal roots in the present study, are a common response during the interaction of tree roots with ECM fungi^20^. Enhanced root surface is most likely beneficial to the plant and fungus, increasing the potential for the uptake of fungal-derived nutrients by the plant and of plant-derived carbon by the fungus.

Our untargeted metabolic analysis confirmed that the metabolome of mycorrhizal oak roots is significantly different from that of non-inoculated roots. Since in our experiment the mycorrhizal roots had only an estimated 7% of *P. tinctorius* biomass we assumed that alterations are most likely occurring in the plant tissues. However, additional experimental verification would be needed to unequivocally prove the origin of the identified metabolites, such as prior isotopic labelling of *P. tinctorius* tissues followed by tracing of labelled fungal metabolites in the mycorrhizal roots^14^. Nevertheless, since most metabolites detected in the cork oak roots were decreased by *P. tinctorius* interaction, they are most likely plant metabolites.

We found that symbiosis with *P. tinctorius* decreased the levels of many metabolites in the mycorrhizal cork oak roots, in agreement with metabolomics studies by Wong et al.^14^, which showed a general repression of metabolites at the pre-mycorrhizal phase of the interaction of *Eucalyptus grandis* and *Pisolithus microcarpus*. More suppressed metabolites were associated with *P. microcarpus* strains with high root colonization potential, suggesting that decreased metabolic responses during the pre-symbiotic early stages could be crucial for successful ECM colonization at latter stages^14^. These results might suggest a common strategy of *Pisolithus* species to colonize the roots of their host plants.

Our results show that *P. tinctorius* was negatively associated with the level of several primary metabolites in the cork oak mycorrhizal roots, such as putative carbohydrates and fatty acids, which content was reduced. Likewise, secondary compounds putatively related to hydrolysable tannins were also reduced following *P. tinctorius* mycorrhization. In contrast, putative non-proteogenic amino acids, like GABA, and some putative secondary metabolites, such as terpenoids and oxylipins, had increased levels in mycorrhizal roots. Previous work on other ECM biological systems have also showed an extensive metabolic reprograming induced by mycorrhiza formation, affecting primary and secondary metabolism^12,13^, derived from the alterations at the transcript and protein levels, detected in several studies^21^.

It must be mentioned that the experiment present here, using young seedlings, does not fully express the endogenous rhythmic growth of oak tress which alternates between shoot and root flushes, that are related to parallel shifts in above- and below-ground allocation of carbon compounds^22^. Since we collected roots during the root growth period of oak seedlings (May–June)^23^, our experimental system might only have a parallel in the root flush period of older seedlings^23^, when carbon allocation is shifted to the roots^24^. The use of oak microcuttings would enable to surpass this limitation and examine the metabolic responses during the whole growth cycle, but these are only available for *Quercus robur*^25^.

Our NMR and FT-ICR analysis showed a reduction in the levels of putative compounds related to sugar and organic acid metabolism in mycorrhizal roots. Besides compounds putatively corresponding to sucrose and glucose, other putative carbohydrates involved in the biosynthesis of sugar alcohols (polyols), like quercitol and sorbitol, were less abundant in mycorrhizal roots when compared to non-inoculated controls. We also detected lower levels of some organic acids, such as lactate and malate, and of the amino acid glycine-betaine. All these compounds have been identified in other studies as components of plant root exudates or associated to apoplastic protective barriers such as the bark tissues of tress^26–28^. Plants exude a considerable proportion (20–40%) of their assimilates^29^, including sugars, amino acids, and organic acids which are believed to be passively lost from the root and used by rhizosphere-dwelling microbes^30^. It has long been acknowledged that carbon compounds in root exudates contribute to attract symbionts in the rhizosphere, like mycorrhizas and beneficial bacteria, that depend on this carbon supply for their survival^29,31,32^. ECM fungi, like *P. tinctorius* associate to the apoplast of roots where these compounds are most certainly exuded. Their decreased levels in the mycorrhizal roots, detected in our experiment, might indicate its transference to the fungus. Decreases in carbohydrates and organic acids were also detected in other ECM interactions^12^ and in previous studies on the *Q. suber* – *P. tinctorius* root symbiotic system^7^. Several of the identified compounds, such as those putatively corresponding to quercitol and glycine-betaine, are very abundant metabolites in tree species naturally subjected to hot and dry climate conditions, such as *Q. suber*, functioning as protective compatible solutes and osmoprotectors^33,34^. Therefore, their high availability in the roots from this species can be suggested since a growing body of evidence shows that carbon allocation to root exudates is driven by disposal of surplus carbon, exudates containing more of the elements that plants have in surplus^29^. According to Buscot and Herrmann^35^, ECM symbiosis is affected by the endogenous rhythmic growth of oaks, being stimulated during growth flushes that increase C resource availability in the roots (root flush). The positive effect in root biomass we observed in our experimental system is probably related to the stimulation of *P. tinctorius* symbiosis during root flush when plant C resources allocated to the roots can be exchanged by fungal derived mineral nutrients, improving plant growth^36^. On the other hand, the decrease in carbohydrate levels detected in the mycorrhizal cork oak roots (relative to non-inoculated roots) is consistent with a sink effect exerted by *P. tinctorius* in order to assimilate part of the C resources accumulating in the roots during root flush. However, under our experimental conditions we cannot confirm that the identified C metabolites are being exuded by roots or used by *P. tinctorius*. On way to address this issue would be by isotopic tracing using those metabolites, combined with transcriptomic analysis to show that pathways leading to those compounds are not being repressed by the symbiotic interaction.

One notable target of the metabolic reprograming induced by *P. tinctorius* in cork oak roots was at the level of lipid metabolism. A decrease in putative monoacylglycerols and fatty acids was observed in the mycorrhizal roots. Our results contrast with those reported in arbuscular mycorrhizas (AM), where the plant activates lipid biosynthesis, transferring fatty acids to the symbiotic fungus^37^. AM fungi are obligate biotrophs that depend on the plant partner not only for carbon supply but also for lipids, with several important genes needed to synthesize fatty acids being absent from the AM fungi genome^38^. However, ECM fungi have a more flexible lifestyle, living in association with plant host roots but surviving also in decaying soil organic matter. Studies have shown that ECM fungi have a complete gene repertoire for fatty acid synthesis, modification and degradation^39^ suggesting an independence from the plant host lipid metabolism. Most of the putative fatty acids detected as down-accumulated in the cork oak mycorrhizal roots, such as isomers of nonadecanoic acid, hexadecanoic acid, hydroxydocosanoic acid, tricosanedioic acid and hexadecanedioic acid are long-chain saturated fatty acids. Remarkably, these class of fatty acids, together with monoacylglycerols (which were also decreased in our experiment), are major components of the suberin and cutin of bark, a layer that accumulates in the outermost face of shoots and roots of trees, protecting tissues from desiccation and pathogen attack^27,40,41^. Other compounds detected in lower levels in the cork oak ECM roots, such as those putatively corresponding to tannins, are also common components of suberin and bark tissues^27,41^. These results, pointing to a decrease in metabolites putatively involved in root protective bark layers are in line with the alterations in plant gene expression consistent with a cell wall loosening process that have been reported in several studies on ECMs^6,42^. This might contribute to facilitate fungal progression in the plant apoplast and symbiosis establishment.

Alterations in compounds putatively involved in the oxylipin pathway were detected in the mycorrhizal roots in our experimental system. Compounds in this pathway, such as jasmonates (JA), are associated to plant defense against pathogens. Increases in oxylipins, such as traumatic acid and azelaic acid, were also detected in ECM roots of *Quercus aliena* with *Tuber indicum*^13^, which may indicate a common response of oaks to ECM fungi. Other compounds putatively involved in response to biotic and abiotic stress detected in the mycorrhizal cork oak roots, matched putative compounds in the terpenoid and flavonoid category. Studies have shown that plant colonization by symbiotic organism such as ECM fungi enhances the production of flavonoids and terpenoids in the host roots^14^, mycorrhizal plants having generally reduced attacks by root-feeding insects^43^. In this context, a recent metabolomics study by Almeida et al.^44^ identified flavonoids and terpenoids as metabolites involved in the ability of cork oak to cope with drought stress, which suggests a common metabolic response to symbiosis and abiotic stress, also detected in other studies^2^. ECM cork oak roots showed increased levels of a compound putatively corresponding to pyridoxamine, a vitamin B6 pathway compound described to participate in mycorrhizal-induced abiotic stress tolerance in tomato plants^45^, and in mycorrhiza induced resistance (MIR) against pathogens^46^.

Increasing evidence support a role of GABA in protecting plants against major abiotic and biotic stressors^18^. Previous studies using RNAseq suggest an increase in GABA synthesis on ECM roots of cork oak with *P. tinctorius*^6^. Therefore, to investigate in deeper detail the role of GABA increases detected in our ECM system we analysed the expression of cork oak genes involved in the GABA shunt, the major pathway leading to GABA synthesis and catabolism in plants^18^. The increased levels of glutamate decarboxylase (GAD1) transcription in mycorrhizal roots, while GABA transaminase (GABAT) expression was reduced, shows that reactions leading to GABA production are being activated, while those associated to GABA catabolism are impaired. The expression of polyamine oxidases (PAO), which can be oxidized to GABA^18^ was increased in the *P. tinctorius* mycorrhizal cork oak roots most likely also contributing to GABA synthesis in this tissue. Studies have found that increases in GABA during pathogenic interactions in plants can be derived from fungi^47,48^. However, this is unlikely in our experiment, since *P. tinctorius* GAD transcription was barely detected in mycorrhizal roots. Though important gaps of knowledge remain to be filled in this sense, there are several reports suggesting the involvement of GABA and related compounds in other symbiotic interactions^49^ including AM interactions, such as the increased GABA levels and GAD activity in mycorrhizal tomato roots^50–52^, or the recent discovery of a *Pisolithus albus* effector which enhances the biosynthesis of polyamines, favouring the colonization of the host *Eucalyptus grandis*^53^. However, to validate the role played by GABA in the ECM symbiosis between cork oak and *P. tinctorius*, additional experimental verification would be needed, such as treatment of cork oak plants with GABA or GABA homologs, followed by evaluation of plant tolerance to abiotic and/or biotic stress. Overall, these results constitute an indication that ECM fungi induce the activation of defense responses, probably to control ECM fungal growth within limits, which might contribute to the priming of defences that protects mycorrhizal plants from pathogenic microorganisms and plant pests^4^. Induction of plant defences by various types of mutualistic organisms including ECM fungi is a common event associated to the recognition of an alien organism^2^. However, even in low compatibility symbiotic interactions (negative effect on plant growth) the fungal partner is recognized as symbiont, and no mechanisms characteristic of the negative pathogenic/parasitic interactions, such as ROS burst or increased lignification, are activated^54^.

Defense responses in mycorrhizal oaks seem to be affected by resource allocation related to the rhythmic growth patterns of the tree, since during the shoot flush the increased sink strength due to dual inoculation (with a nematode and an ECM fungus) results in a suppression of plant defenses, likely a response of the plant to reallocate reserves aboveground^55^. Since we collected roots during root flush, when carbon resources are allocated to roots, a negative effect on plant defense responses related to the sink effect exerted by *P. tinctorius* is not expected, which is in accordance with our results showing the accumulation of compounds putatively involved in plant defense.

In conclusion, using a non-targeted metabolomics approach we were able to characterize the metabolic reprogramming occurring in cork oak roots following ECM formation with *P. tinctorius*. A shift in primary and secondary root metabolism was observed. Several of the metabolic changes detected are putatively related to plant defense and immunity mechanisms and could be involved in the mycorrhizal enhancement of plant stress resistance that has been reported in several studies. In this context, compounds such as GABA, pyridoxamine, or terpenoids have been previously shown to prime plant immunity against pathogen attack, improving also resistance to abiotic stresses in symbiotic plants^45,49^. These are interesting targets for a detailed functional study aiming to investigate their role in ECMs and the associated molecular mechanisms related to improved resistance against biotic and abiotic stresses.

## Methods

### Plant material and *P. tinctorius* inoculation

The *P. tinctorius* strain Pt23 was maintained in pure culture on BAF agar medium containing glucose 1%, in the darkness at 23 °C. *Quercus suber* acorns were surface sterilized with a 10% commercial bleach solution and germinated in trays containing soil acquired from a gardening store (80–150 mg/L N, 80–150 mg/L P2O5, 300–500 mg/L K2O, pH (CaCl2) 5.5–6.5, organic matter > 70%). Soil was autoclaved (121°, 1 atm., 60 min) before use. Four-week old plantlets were transferred to 2L pots containing autoclaved soil and inoculated with *P. tinctorius* inoculum according to Sebastiana et al.^7^. Control plants were treated with an autoclaved *P. tinctorius* inoculum. Plants were maintained in soil in a greenhouse under a randomized block design. A total of 15 plants were used for each treatment. Eight weeks upon *P. tinctorius* inoculation, mycorrhizal and non-inoculated plants were harvested for analysis (May–June). First, the root system of each plant was gently washed with demineralized water. The fresh weight of leaves and roots (whole root system) was determined. Significant differences between mycorrhizal and non-inoculated plants were assessed using T-test (*P *value ≤ 0.05), after testing data for normality using the Shapiro Wilk test and for equality of variance using Levene’s test. An aliquot of 3 individual inoculated and non-inoculated root systems was reserved for mycorrhizal root colonization assessment. Next, lateral roots showing at least one mycorrhiza were cut at the intersection with the principal root with a scalpel blade and immediately frozen in liquid nitrogen for further analysis. In parallel, lateral roots from non-inoculated plants were collected in the same way. At collection, roots from mycorrhizal and non-inoculated plants were visually inspected for the presence of fungal mycelium, besides that of *P. tinctorius.* No signs of mycelium from other fungi were detected. Roots from 3 independent plants were pooled together constituting in one biological replicate. In total, 5–3 biological replicates from mycorrhizal roots and non-inoculated roots were collected for analysis. Each root biological replicate was grounded to a fine powder using liquid nitrogen and stored at − 80 °C.

### Root ergosterol concentration and fungal biomass

Root ergosterol concentration was determined in roots using 3 biological replicates, each composed of a pool of 3 individual root systems, and in *P. tinctorius* pure culture, as described in Sebastiana et al.^7^. Significant differences between *P. tinctorius* inoculated and non-inoculated plants were assessed using Mann–Whitney *U* test (*P *value ≤ 0.05). The % of *P. tinctorius* biomass in mycorrhizal roots was estimated by calculating the ergosterol in mycorrhizal roots (ergosterol in mycorrhizal roots – ergosterol in non-inoculated roots) relative to the ergosterol of a *P. tinctorius* pure culture (100 percentage ergosterol) (Supplementary Table S1).

### Nitrogen root concentration

The nitrogen concentration of roots was determined at the Stable Isotopes and Instrumental Analysis Facility, Faculty of Sciences, Lisbon University. Three biological replicates, each consisting of a pool of roots from 3 plants, were analysed for each treatment. Frozen root material was dried at 70° C for 72 h and grounded in a mill (Retsch, Germany) to a homogenous fine powder for isotopic analysis. After grinding, samples were used for N percentage calculation, according to Rodrigues et al.^56^, on a EuroEA 3000 CHNS-O Elemental Analyzer (EuroVector, Milan), with a TDC detector. Root N concentration was defined as % of dry weight. Significant differences between *P. tinctorius* inoculated and non-inoculated plants were assessed using Mann–Whitney U test (*P *value ≤ 0·05).

### ^1^H NMR analysis

#### ^1^H NMR sample preparation

Freshly collected root samples were immediately packed and frozen in liquid nitrogen. This frozen root material (5 biological replicates from mycorrhizal and non-inoculated roots) was lyophilized, its metabolites were extracted using a mixture of water/methanol and then transferred into NMR sample tubes, as described in Rivas-Ubach et al.^57^. Trimethylsilyl propionic acid (TSP), was used as internal standard.

#### ^1^H NMR fingerprinting

High-resolution ^1^H NMR spectroscopy measurements were conducted using a Bruker AVANCE 600 spectrometer equipped with an automatic sample changer and a multinuclear triple resonance (TBI) probe (all from Bruker, Rheinstetten, Germany) at a field strength of 14.1 T (600.13 MHz ^1^H frequency), following the methodology described in Rivas-Ubach et al.^57^ and Gargallo-Garriga et al.^58^. The determination process was controlled using TopSpin 2.1 software (Bruker, Rheinstetten, Germany).

#### ^1^H NMR data analysis

A bucketing process and peak mean centering and standardization for statistical analyses of the 1D ^1^H-NMR spectra were conducted using AMIX software (Bruker, Rheinstetten, Germany), as described in Rivas-Ubach et al.^57^. Multivariate analyses (PCAs based on correlation matrices) were performed to detect patterns of sample ordination according with the metabolic profile. Differences in PCA scores between mycorrhizal and non-inoculated roots were tested by one-way ANOVA. Differences in individual peaks (integral values of each metabolite) between mycorrhizal and non-inoculated roots were determined using T-test. The normality of each variable was tested by Kolmogorov–Smirnov test. All variables followed normal distributions. Statistical analyses were performed by using vegan, mixOmics, nlme, ggplo2, ade4 and FactoMineR packages in R v3.5.1 Core software.

#### ^1^H NMR metabolite identification

The sample extracts were also used for the acquisition of the 2D NMR sampling spectra. The metabolite identification was done as described in Rivas-Ubach et al.^57^ based on the structural elucidation of the spectra. Briefly, hydrogens (protons) connected through three to five chemical bonds were identified using 2D ^1^H-NMR homonuclear COSY and TOCSY correlations, whereas ^1^H-^1^H NOESY method was used to determine connections between distinct parts of target molecules. Finally, heteronuclear ^1^H-^13^C HSQC and HMBC methods were used to identify the carbon skeleton of each target molecule. Spectra were referenced to TSP (^1^H and ^13^C at δ 0.00 ppm). When possible, assignments were confirmed with described databases^59–72^.

### FT-ICR analysis

#### FT-ICR sample preparation

Sample preparation and metabolite extraction were performed following the protocol previously described^73^. Briefly, frozen root powder (100 mg) from each sample (3 biological replicates from mycorrhizal roots and non-inoculated roots, and *P. tinctorius* fungal mycelium growing in pure culture) was extracted using 1 ml of solvent mixture (methanol, chloroform and water (2:2:1, v/v/v). Samples were then vortexed (1 min) and maintained for 15 min in an orbital shaker for metabolite extraction. Samples were centrifuged (1000 g, 15 min, room temperature) for phase separation: chloroform fraction and aqueous-methanol fraction. The chloroform (C) fraction was collected, re-centrifuged and lyophilized at -40 °C. The aqueous-methanol fraction was subjected to solid-phase extraction (LiChrolut RP-18columns, Merck) and the metabolites were sequentially eluted (twice) with 1 mL of water and 1 ml of methanol. The water (W) fraction was lyophilized at − 40 °C, while the methanol (M) fraction was evaporated under a stream of nitrogen. The W and C fractions were solubilised in a water/methanol solution (1:1, v/v), whereas the M fraction was solubilised in the respective pure solvent.

#### FT-ICR mass spectra acquisition

Each sample was diluted 1000-fold in the appropriate solvent: M fraction was diluted in the same solvent for positive- (ESI^+^) and negative-ion (ESI^−^) mode analysis; W and C fractions were diluted in methanol for ESI^+^ and in methanol/water (1:1) for ESI^−^. Leucine enkephalin (YGGFL, Sigma Aldrich) was added to all the fractions (0.5 μg/mL) and was used for internal calibration and for quality assessment of analytical precision ([M + H]^+^  = 556.276575 Da or [M-H]^−^  = 554.260925 Da). The diluted extracts from each sample were analysed by direct infusion in an Apex Qe 7-T Fourier Transform Ion Cyclotron Resonance Mass Spectrometer (FT-ICR-MS, Bruker, Bremen, Germany). Spectra were acquired using electrospray ionization (ESI) in positive (ESI^+^) and negative (ESI^−^) modes, as described previously^73^. Mass spectra were recorded with an acquisition size of 512 k, in the 100 to 1000 m/z mass range^73^.

#### FT-ICR data analysis

Mass spectra were analysed using the software package Data Analysis 5.0 (Bruker, Bremen, Germany). Single point calibration was performed with the leucine enkephalin standard, both in positive and negative ionization modes. Mass peak lists were exported as ASCII files, with a signal-to-noise ratio of 4. Data Analysis 5.0 was also used to obtain putative assigned compound formulas. Smart formula tool was used following the upper formula: C_78_H_126_O_27_P_9_S_14_N_20_ and lower formula: C_1_H_1_O_0_P_0_S_0_N_0_ as described in Kind and Fiehn^74^. Formulas were exported to plot H/C ratios against O/C ratios for mycorrhizal and non-inoculated roots (Van Krevelen diagrams). Peak lists of the different samples were aligned into a single matrix with a maximum error of 2.0 ppm deviation between replicates using an in-house Python script, as previously described^73^. Only the ions detected in at least 2 biological replicates were considered. Features with 3 intensity values or 3 missing values in at least one of the treatments were selected for further analysis. The acquired data matrices were further analysed with the MetaboAnalyst (4.0) package^75^ (https://www.metaboanalyst.ca/). Peak intensities were median normalized, log transformed, and Pareto scaled. A multivariate data analysis approach was performed using Partial Component Analysis (PCA) and Principal Least Square Discriminant Analysis (PLS-DA). A PCA was applied to examine global metabolite variation triggered by the two treatment groups: mycorrhizal inoculation and control. Then, a PLS-DA was used to classify the groups segregation and identify the most important metabolites explaining the changes in metabolic profiles between treatments. Metabolites with variable importance in projection values (VIP) > 1 were selected as the most important features having the highest discriminating capacity between the treatments.

#### FT-ICR metabolite identification

The putative identification of metabolite identities was conducted by searching against chemical public databases, such as Kyoto Encyclopaedia of Genes and Genomes (KEGG), Human Metabolome Database (HMDB) and LIPID MAPS, using the MassTRIX interface^76^ (http://masstrix.org). The final mass list was uploaded to the MassTRIX 3 server with the following parameters: scan mode was either positive or negative; adducts considered were M + H, M + K and M + Na for ESI^+^ data and M-H and M + Cl for ESI^−^ data; maximum *m/z* deviation was 3 ppm; search was performed in the “KEGG/hmdb/LIPID MAPS without isotopes” databases. Metabolite annotation was further accomplished using the annotation pipeline developed by Nascimento et al.^77^. Search results matching to synthetic, bacteria- or animal-derived compounds were discarded. For compounds with multiple annotations a manual curation was performed, and the putative metabolite classes were described based on the common chemical structure.

### Analysis of gene expression by qPCR

The expression of genes involved in gamma-aminobutyric acid (GABA) synthesis and degradation was analysed by qPCR. In order to design cork oak specific primers, the following *Arabidopsis* protein sequences encoding GABA metabolic enzymes were used for searching the NCBI database (TBLASTN) restricted to *Quercus suber* (taxid: 58,331): GAD1 (AT5G17330.1), GABAT (AT3G22200.2), PAO4 (AT1G65840.1), and PAO2 (AT2G43020.1). A *P. tinctorius* GAD mRNA sequence annotated as glutamate decarboxylase (jgi|Pisti1|18,961|gm1.1382_g) was retrieved from the JGI database and used for specific primer design. Total RNA from mycorrhizal and non-inoculated roots was extracted using the protocol of Wan and Wilkins^78^. Total RNA was treated with DNase using the Turbo DNA-free kit (Invitrogen) and first-strand cDNA was synthesized from purified total RNA using Reverse Transcriptase (RevertAid H Minus M-MuLV Reverse Transcriptase, Fermentas) and Oligo dT primer. Three biological replicates (composed of roots from 3 independent plants each) were analysed per treatment. qPCR was performed as described previously^79^. Information on genes, primer sequences and qPCR amplification are shown in Supplementary Table S5. Melting curve analysis was used to identify non-specific PCR products (Supplementary Fig. S3). Relative quantification of specific transcript levels was calculated using the Livak and Schmittgen method^80^. Expression values were normalized using the cork oak elongation factor-1α gene (EF-1α), previously reported as a reference gene in cork oak-*P. tinctorius* ECMs^7^. Significant differences between mycorrhizal and non-inoculated roots were assessed using Mann–Whitney *U* test (*P *value ≤ 0.05).

## Supplementary Information

Supplementary Information 1.

Supplementary Information 2.

Supplementary Information 3.

Supplementary Information 4.

Supplementary Information 5.

Supplementary Information 6.

Supplementary Information 7.

Supplementary Information 8.

Supplementary Information 9.

## Data Availability

The datasets generated during the current study are available in figshare data repository with the identifiers https://doi.org/10.6084/m9.figshare.13379852.v1^81^ and https://doi.org/10.6084/m9.figshare.14096213.v1^82^.
