# Supplementary material for: Metabolomics and transcriptomics to decipher molecular mechanisms underlying ectomycorrhizal root colonization of an oak tree
Source: Sci Rep. 2021 Apr 21;11:8576. doi: 10.1038/s41598-021-87886-5 (PMC8060265; doi:10.1038/s41598-021-87886-5)
Supplement: Supplementary file 1 — Supplementary Information 1. [file 41598_2021_87886_MOESM1_ESM.docx]

**Metabolomics and transcriptomics to decipher molecular mechanisms underlying ectomycorrhizal root colonization of an oak tree**

**M Sebastiana, A Gargallo-Garriga, J Sardans, M Pérez-Trujillo, F Monteiro, A Figueiredo, M Maia, R Nascimento, M Sousa Silva, A N Ferreira, C Cordeiro, A P Marques, L Sousa, R Malhó and J Peñuelas**

**Supplementary material**

Supplementary Table S1 – Leaf biomass and root N concentration of mycorrhizal (Myc) and non-mycorrhizal (Cont) cork oak plants. Ergosterol concentration and % of *P. tinctorius* biomass in mycorrhizal roots.

Supplementary Table S2 - ^1^H and ^13^C NMR assignments of the major metabolites identified in polar extracts [CD_3_OD-D_2_O 1:1 (pH 6.0)] of roots. Chemical shifts are referenced to TSP.

Supplementary Table S3 – Number of peaks detected by FT-ICR in ESI+ and ESI- ionization mode per biological replicate of mycorrhizal (Myc) roots and non-mycorrhizal (Cont) roots.

Supplementary Table S4 – Supplementary Table S4 - List of annotated discriminant metabolites differentiating mycorrhizal from control roots obtained in positive (ESI+) and negative (ESI-) ion analysis modes. Raw mass is the mass detected by FT-ICR-MS; the neutral corrected mass (without adduct), the mass in KEGG database (with adduct) and the deviation (in ppm) are indicated. The different putative metabolites, as well as their mass in KEGG, deviation (in ppm), ID (in KEGG,HMDB,LipidMaps) and formula, are presented and separated by a #. VIP score (VIP>1) and log2(FC) are indicated (log2(FC) represents the fold change between myc and control groups). The chemical formula of the compounds and the putative identification with compound taxonomy (Major Class and Secondary Class) are presented. Compounds identified in *P. tinctorius* mycelium growing in pure culture are identified by (+).

Supplementary Table S5 – Genes, primers and amplification data of the qPCR analysis. Gene accession numbers, primer sequences (Fw, forward; Rev, reverse), amplicon length, primer annealing (Ta) temperature and amplification efficiency are indicated. ^a^ Alternative splicing variants.

Supplementary Figure S1 - ^1^H NMR metabolic profile of the polar (water–methanol 1:1) extract sample of roots. Assignments of the main peaks are indicated. The sample was dissolved in CD_3_OD-D_2_O 1:1 (pH 6.0) and referenced to TSP. Spectra were acquired at a magnetic field of 600 MHZ and at 298.0 ºK.

Supplementary Figure S2 **–** van Krevelen plots showing the positioning of the various classes of metabolites in mycorrhizal roots (a) and control roots (b). The metabolite profiles are derived from FTICR-MS analysis.

Supplementary Figure S3 – Melting curves of the qPCR analysis of the reference gene (*EF1α*) and target genes (*GAD1*, *GABAT*, *PAO2*, *PAO4* and *PtGAD2*).
