# Supplementary figures and images for: Metabolomics and transcriptomics to decipher molecular mechanisms underlying ectomycorrhizal root colonization of an oak tree
Source: Sci Rep. 2021 Apr 21;11:8576. doi: 10.1038/s41598-021-87886-5 (PMC8060265; doi:10.1038/s41598-021-87886-5)

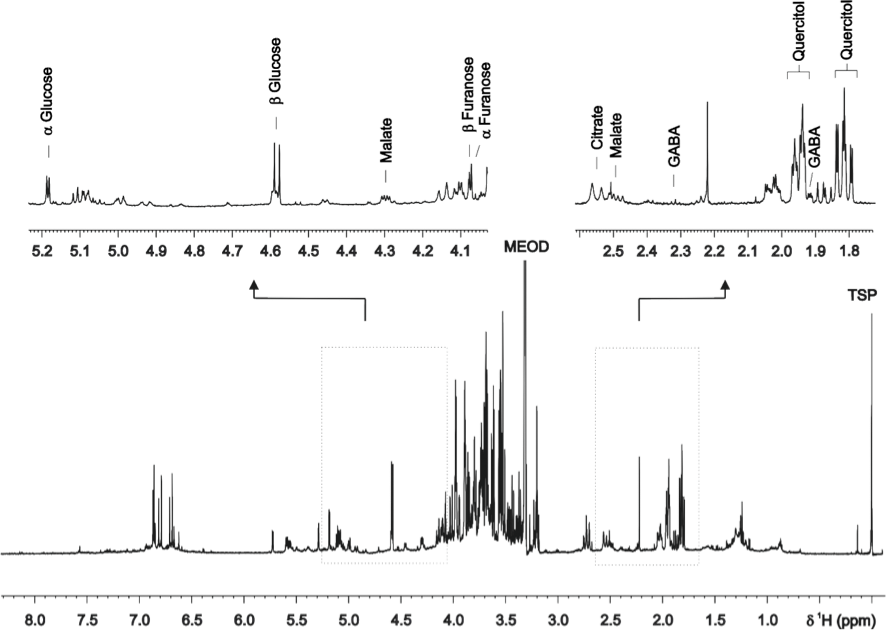

Supplement: Supplementary file 2 — Supplementary Information 2. [file 41598_2021_87886_MOESM2_ESM.tif]

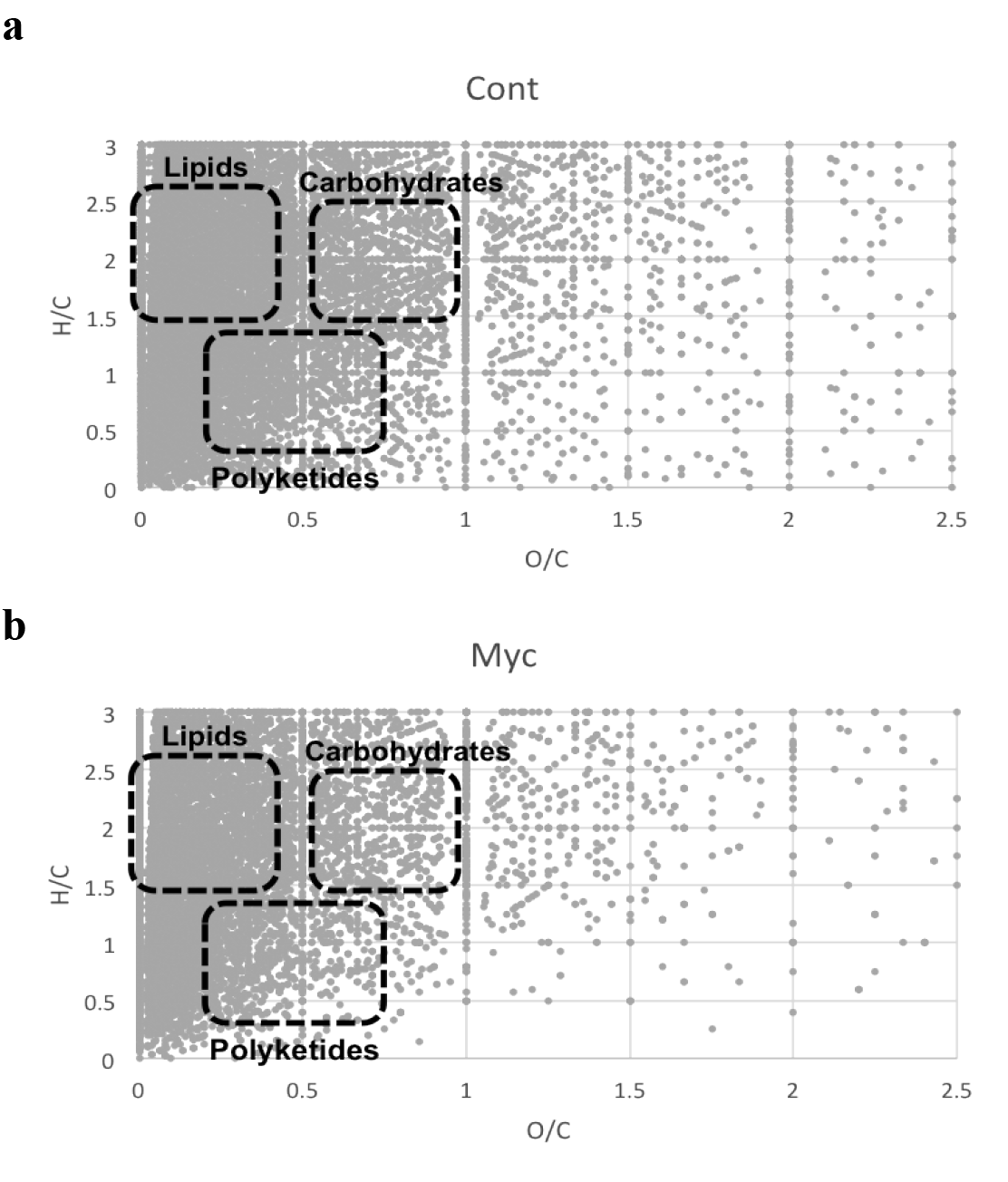

Supplement: Supplementary file 3 — Supplementary Information 3. [file 41598_2021_87886_MOESM3_ESM.tif]

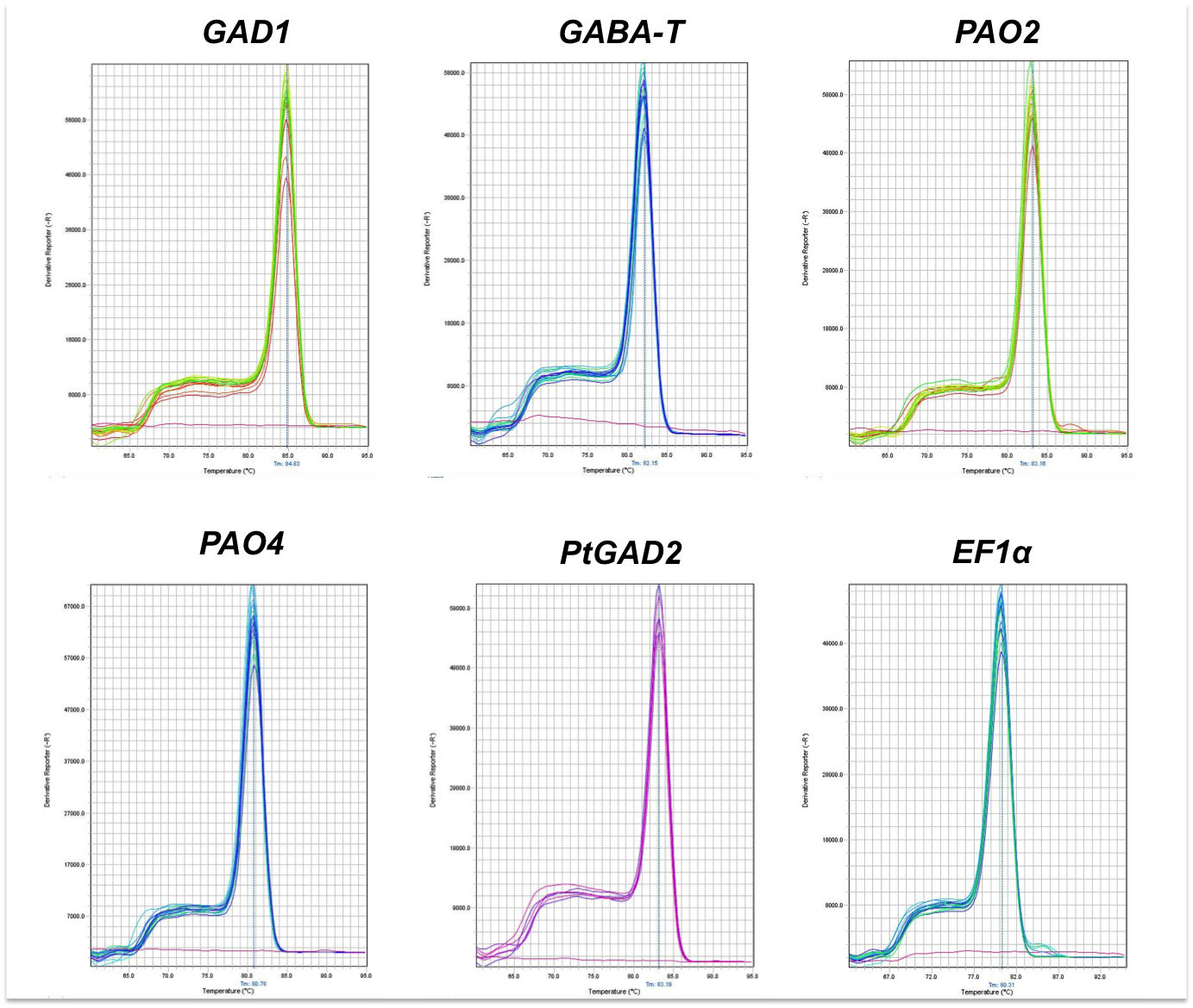

Supplement: Supplementary file 4 — Supplementary Information 4. [file 41598_2021_87886_MOESM4_ESM.tif]
