# Supplementary material for: Metabolomics and transcriptomics to decipher molecular mechanisms underlying ectomycorrhizal root colonization of an oak tree
Source: Sci Rep. 2021 Apr 21;11:8576. doi: 10.1038/s41598-021-87886-5 (PMC8060265; doi:10.1038/s41598-021-87886-5)
Supplement: Supplementary file 6 — Supplementary Information 6. [file 41598_2021_87886_MOESM6_ESM.docx]

**Supplementary Table S2** - ^1^H and ^13^C NMR assignments of the major metabolites identified in polar extracts [CD_3_OD-D_2_O 1:1 (pH 6.0)] of roots. Chemical shifts are referenced to TSP.
